# Acoustic emission of lattice structures under cycling loading relates process parameters with fatigue properties

**DOI:** 10.1038/s44172-024-00196-2

**Published:** 2024-03-22

**Authors:** Lea S. Kollmannsperger, Oliver Maurer, Rebecca Kose, Andre T. Zeuner, Dirk Bähre, Sarah C. L. Fischer

**Affiliations:** 1 Fraunhofer Institute for Nondestructive Testing IZFP, Campus E3.1, 66123 Saarbruecken, Germany; 2 Institute of Production Engineering, Saarland University, Campus A4.2, 66123 Saarbruecken, Germany; 3 Centre for Mechatronics and Automation Technologies (ZeMA), Eschberger Weg 46, 66121 Saarbruecken, Germany; 4 Fraunhofer Institute for Material and Beam Technology IWS Winterbergstr. 28, 01277 Dresden, Germany

**Keywords:** Mechanical engineering, Mechanical properties, Characterization and analytical techniques, Metals and alloys

## Abstract

Metamaterials, especially lattice structures, are of great interest for many application areas such as aerospace, automotive and medicine due to their adjustable mechanical properties and their low weight. Due to their complex geometry, lattice structures are usually manufactured additively, which causes a large variance in the manufacturing-related mechanical properties. In order to establish metamaterials in industrial applications under cyclic loading, the fatigue behavior needs to be investigated to evaluate the load capacity of these structures. Here we analyze the fatigue behavior of AlSi10Mg truss structures fabricated with L-PBF using a load increase test in combination with acoustic emission measurements. The acoustic signals are evaluated in terms of time-dependent amplitude signal and frequency spectrum. Increasing load and increasing specimen damage resulted in changes of the acoustic spectrum and the amplitude of the time signal. Based on the results, a correlation of specimen properties with build platform position in the manufacturing process could be established. Acoustic emission measurement as an in situ characterization method during cyclic loading is promising for surveillance of lattice structures in safety related applications.

## Abstract (web summary)

Lea Kollmannsperger and colleagues report the fatigue behaviour of additively manufactured lattice structures under cyclic loading, relating fatigue-induced properties to process characteristics using acoustic emission. The results can help to predict damage during operation through non-destructive testing.

## Introduction

In times of rising material and energy costs, the main focus of research and industry is on identifying and developing savings opportunities. Metamaterials, and in particular lattice structures, offer a promising approach to realize lightweight design concepts that save material without compromising structural stiffness^1,2^. The properties of metamaterials are mainly determined by the microstructural architecture^3,4^. By varying the strut thickness, strut length, material, or number of unit cells, the mechanical properties of the lattice structure can be specifically adjusted^1,5–7^. Due to the complex geometry, lattice structures are usually manufactured by additive manufacturing processes such as Laser Powder Bed Fusion (L-PBF). For this purpose, metallic specimens are manufactured layer-by-layer based on a CAD model by distributing metallic powder on the building platform and subsequent melting by means of a laser beam^8–10^. Due to the variety of adjustable manufacturing parameters, such as the choice of support structues^11–13^, scan strategy^14–17^, or scanning speed^18–22^ in the L-PBF process, the identification of parameters suitable for the part geometry is very complex^23,24^. It may even be necessary to perform post-processing steps, such as heat treatment^25–27^, to achieve the required application properties. Additive manufacturing processes are known to create certain types of defects^28^. This includes pores, melting faults, and a surface topography that is characterized by a pronounced surface roughness as well as downskin and upskin effects^21,29,30^. A suitable choice of support structures, which specifically adjusts the angle of the specimen to be produced relative to the incident laser beam, makes it possible to minimize adhesion to the surface and the downskin and upskin effects^11–13,31^. Although it is well known that the quality and properties of specimens depend to a considerable degree on the manufacturing parameters, most previous studies, due to the relatively low effort involved, deal with the characterization of mechanical properties by means of quasi-static material testing. Here, it is primarily volume properties that are evaluated. Lattice structures show a characteristic response towards uniaxial compressive loading, consisting of three domains: a linear elastic domain, a plastic deformation domain, and a material densification domain^1,5,32–34^. Maskery et al.^32,35^ were able to identify successive cell collapses, propagation of cracks through lattice and diagonal shear as sources of failure. In real industrial applications, dynamic, cyclic as well as multi-axial loads occur, which is why a characterization of lattice structures under these loads is essential for the design of specimens. In the field of mechanical damping applications, strain rate-dependent mechanical properties in additively manufactured lattice structures are of great interest. In their studies, Lee et al.^36^ found strain rate sensitivity of mechanical properties in stainless steel lattices. While studies by Liu, Hou^37^ and Tang^38^ could attribute an increase in dynamic strength to inertial effects. Mc Kown^39^ and Thomas^40^ considered that this effect was due to the rate sensitivity of the base material. Xiao et al.^41^. Found a strain rate sensitivity depending on the lattice architecture. Studies on strain rate-dependent acoustic damping properties of metamaterials have also been performed. Here, different acoustic damping ranges depending on the strain could be identified by Babaee et al.^42^. Complementary cyclic tests, which complete the mechanical characterization, are often neglected due to the high effort involved. While the additively manufactured bulk material is already well characterized^11,15,29^ and thus the influencing factors are known, only a few research groups deal with the cyclic stress of the metamaterials, although the defects play a major part due to the surface-to-volume ratio^43–45^. Simulations based on quasi-static tests are the tool of choice to assess cyclic damage behavior. For lattice structures, A. Zargarian et al.^46^ were able to determine a three-stage specimen response even under cyclic loading. In the first region, the load increases sharply and then transitions to the 2nd region, where the cumulative load remains approximately constant. In the 3^rd^ region, an exponential increase in loading is evident, culminating in the failure of the structure. In addition, they were able to identify the relative density and topology of the lattice structures as major factors of the failure. Due to the large number of factors influencing the properties of the structural components, characterization based on conventional standardized fatigue tests is very time-consuming. A very large number of specimens is required for a stochastically oriented characterization. Starke et al.^47^. Were already able to show for bulk specimens that non-destructive evaluation methods (NDE) are a useful supplement to fatigue tests. By combining destructive and non-destructive material testing, it is possible to greatly reduce the effort required to evaluate cyclic damage behavior. One NDE already used for damage measurement is the acoustic emission measurement^48^. This method is mainly used to detect crack propagation in fiber composites, concrete^49^, rock^50^, or solid metallic materials^48^. The acoustic emission of the specimen during loading is recorded and analyzed. An approach for application to lattice structures was developed by Y. Ibrahim et al.^51^. Three-point bending tests were performed, accompanied by measurement of acoustic emission. The acoustic signals were analyzed in terms of excited frequencies. Based on their research, they were able to determine that acoustic testing can be used as a measurement method to determine loose powder adhesion to the sample and associated mechanical properties such as relative specimen density and effective modulus. Another approach to the use of acoustic emission measurement was shown by Drissi-Daoudi^52^ and Zhirnov^53^, who used acoustic emission measurement to identify stress cracks developing in the L-PBF process as in situ process monitoring. The previous work shows that there are promising approaches to characterize the fatigue behavior of specimens, but they should be further developed for more complex specimen geometries. Also, the listed studies on acoustic emission measurement represent a promising approach to complement the characterization of the fatigue behavior of structures in a meaningful way. Based on this, the fatigue behavior of additive-manufactured lattice structures is studied in this paper, and a method for determining the damage during loading is developed. This will provide novel insights for the translation of metamaterials to industrial applications, where a deep understanding of their cyclic properties will be essential. AlSi10Mg lattice specimens were fabricated by L-PBF and characterized as a function of their position on the build platform. Based on five quasi-static compression tests in combination with a numerical simulation, cyclic load increase experiments were designed. Load increase experiments and accompanying acoustic emission measurements are combined with damage accumulation modeling to deepen understanding of the complex interplay between manufacturing parameters as well as geometrical parameters.

## Methods

### Specimen production by L-PBF

All the specimens were produced on an SLM125 L-PBF-machine by SLM Solutions Group AG (Lübeck, Germany) from AlSi10Mg-powder with a particle size distribution from 20 µm to 63 µm by the same provider, and the chemical composition^54^ summarized in Table 1.

**Table 1:** Chemical composition of the AlSi10Mg-powder, according to manufacturer^54^

Element	Al	Si	Fe	Mg	Mn	Ti	Zn	Others each
Wt.%	Bal.	9-11	0.55	0.45	0.45	0.15	0.10	0.05

**Fig. 1:**
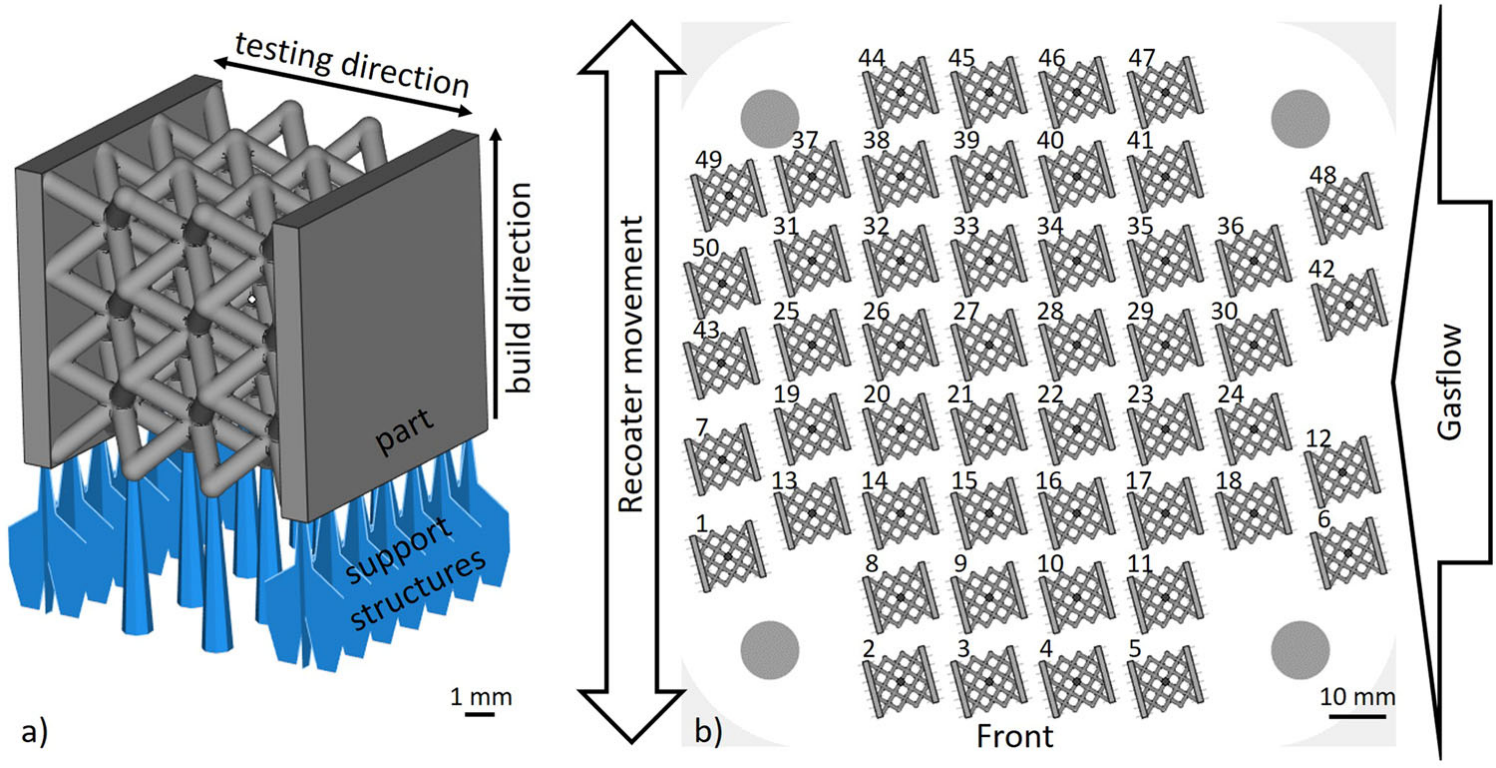
Specimen geometry and position on the build platform. **a** L-PBF-sample geometry including support structures, **b** alignment of 50 samples on a 125 mm × 125 mm substrate plate (white, where the light gray shading highlights the rounded corners and the dark gray shaded circles in the four corners of the building platform representing the mounting holes), recoater movement and gas flow direction.

For the study, a bcc-shaped lattice structure with a strut diameter of 0.75 mm, a strut length of 2.9 mm, and a cell size of 2.5 mm, was constructed in an arrangement of 3 × 3 × 3 cells. They were designed with two plates on the faces with dimensions 10 mm × 10 mm × 0.5 mm in Netfabb Premium 2023 (Autodesk Inc., San Rafael, CA, USA) (Fig. 1a). Here, the specimen geometry represents a compromise between complex structure and good describability, thus offering the possibility to take a step towards analyzing the fatigue behavior of metamaterial structures. The number of unit cells was selected in such a way that with efficient use of materials, a representative lattice with nodes not connected to the plates was obtained. The combination of the lattice structure with the plates, which are intended to apply uniform forces to the specimen, results in an overall size of 10 mm × 10 mm × 11 mm per specimen. In addition, the design created using the software provides the ability to prepare the exact same geometry for both a build job and simulations.

Parameter assignment, sample designation, and positioning on the substrate (see Fig. 1b), as well as slicing and integration of support structures, were carried out by means of Magics software (Materialise NV, Leuven, Belgium). Support structures were used to realize the lattice structure while manufacturing and to provide thermal conduction between the sample and the substrate. Inside the lattice structures, support structures were omitted due to the lack of removability. In order to avoid deterioration of part quality due to large amounts of heat flowing through the lattice, base plates were attached to the sidewalls of the geometry, where they got their own line supports. Most essential process parameters are listed in Table 2. Total fill describes the scanning strategy in which parameters and scan paths of the contour section are scanned in nested lines from outside to inside until an entire irradiation area is completely scanned. This aims to improve the physical properties of lattice struts compared to the usual contour and hatch strategy.

**Table 2:** Key process parameters for the manufacturing of the lattice structure samples

Parameter	Powder layer thickness	Laser power	Scan speed	Hatch distance	Linear energy density	Scan strategy	Shielding gas
Unit	µm	W	mm s^−1^	Mm	J mm^−1^	-	-
Value	30	250	2000	0.1	0.125	Total fill	Argon

After a heat treatment (2 h at 300 °C (573 K)) was performed according to the machine manufacturer’s recommendation^54^ to improve the ductility of the rather brittle initial condition, the samples were cut off the substrate, and the supports were removed. No further post-processing steps were applied. Consequently, surfaces of lattice and base plate sections remained in the as-built state regarding roughness and porosity.

### Archimedes density measurement

To determine the relative density and mass of each sample, the analytical balance AT200 from Mettler (measuring accuracy of ± 0.15 mg) was used. The mass of the sample in air was determined, followed by the measurement of the sample in water. The measurements were performed at a water temperature of 18.6 °C. For statistical validation, each sample was measured three times.

### Quasi-static compression tests

To determine the quasi-static mechanical properties of the designed structures, a quasi-static compression test was performed on six lattice specimens. For this purpose, the tensile testing machine DOLI INSTRON 851120 was used. The test was performed at a strain rate of 0.005781 s^−1^, a maximum displacement of 5 mm and a room temperature of 23 °C. For each of the six specimens, Young’s modulus was calculated from the slope of the obtained stress-strain curve. In addition, the plastic collapse stress (PCS) were determined^5^.

### Load increase experiments

**Fig. 2:**
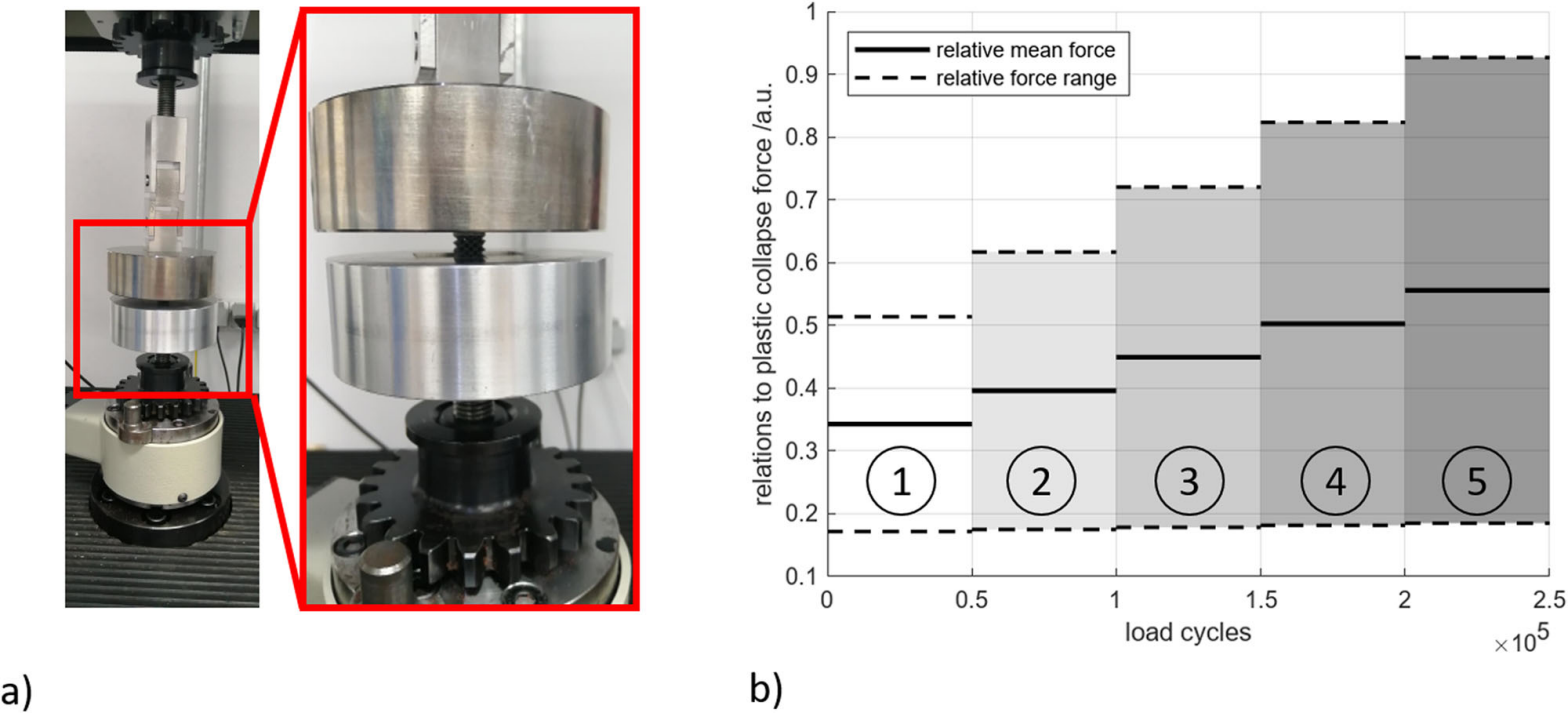
Load increase test. **a** experimental setup in the testing machine with enlarged view of the positioning of the specimen between the two compression plates. **b** Applied load levels 1 to 5, in relation to the plastic collapse stress over load cycles, where the load levels are separated by different gray shading, the relative mean force for each cycle is shown as a straight line and the corresponding real force range is shown as a dashed line.

A 3-mass resonance testing machine (Rumul MIKROTRON, Russenberger Prüfmaschinen AG, Switzerland, see Fig. 2a) was used to determine the cyclic mechanical properties. The specimen was positioned between two plates with a recess in the lower plate. This prevented the specimen from slipping during the experiment. To ensure an even distribution of pressure on the specimen, the upper plate was connected to the plunger via a double universal joint. The loads were measured with a 20 kN load cell positioned below the lower plate. Due to the overall stiffness of the system, the tests could be carried out with a test frequency of ~200 Hz. Based on the quasi-static compression tests, a five-stage load increase test was planned (Fig. 2b) with a stress ratio of *R* = 3 used for the tests. For load level 1 the maximum compressive load was 50% of the PCS, whereas for load level 5 the maximum force achieved 90% of the PCS and ensured the specimens to fail. In between, three intermediate stages were defined equidistantly (stage 2 = 60%, stage 3 = 70%, stage 4 = 80%), resulting in a linear load increment test. Each load level was applied for 5 × 10^5^ load cycles. A change in the resonance frequency of ± 10 Hz was defined as the termination criterion.

### Comsol-simulation

Numerical simulations were performed on 3D-models extracted from Netfabb Premium (see Fig. 1a). The boundary conditions were applied at the top and bottom surfaces of the plates and selected as such that translational movement was prohibited in all directions, whereas rotations were allowed. This is equivalent to the boundary conditions of the associated experiment. Assuming small deformations, a linear elastic material model without plastic deformation was applied. The selected Young’s modulus of 62.27 GPa was measured in previous uniaxial tension experiments on single struts, produced with the manufacturing parameters listed in Table 2. The measurements were performed with an extensometer to account for machine compliance. The density was assumed as 2.58 g cm^−3^, which corresponds to 93% of the theoretical powder density and corresponds to the maximum density capacity of the sintering process^54^. For the finite element calculation a tetrahedron mesh (max. element size = 0.153 mm, min. element size = 0.002 mm, number of elements: 1,075,268) with linear interpolation was chosen, which represents a compromise between calculation time and accuracy. A convergence study was performed based on the change in reaction force. The mesh size selected here deviates from the convergence point by 0.7%. This results in an error margin of +− 1% for the illustrated stress ratios. In a first study, a quasi-static uniaxial compression simulation was performed with the solid mechanics module in Comsol Multiphysics® (version 6.0). A displacement of 25 µm was imposed to the top plate to visualize the relative resulting stress distribution. By choosing the above meshing parameters, the compromise between accuracy of simulation and reduction of computational time led to minor numerical errors when estimating the relative stress concentrations. In a second study, natural frequencies of the structure were simulated with the prior described boundary conditions using the Solid Mechanics physical interface in the Structural Mechanics module in Comsol Multiphysics® (version 6.0).

### Acoustic emission monitoring

The load increase tests were accompanied by acoustic emission monitoring. For this purpose, a custom-made setup consisting of a phantom power supply (Millenium PP2B power supply, phantom power), a signal recorder (U-Phoria UMC404HD, Behringer, Germany), a microphone (measurement microphone ECM8000, Behringer, Germany) and a laptop was used. The microphone was located in front of the structure. The measurement signals were recorded with the free audio editor and recorder software Audacity (das Audacity Team, Germany). The maximum sampling rate allowed by the sound emission system was 96 kHz. According to the Nyquist rule, the maximum resolvable frequency was 48 kHz. Room temperature and humidity were measured using a commercially available thermo-hygrometer (COSY BARO, TFA DOSTMANN, Wertheim-Reicholzheim, Germany). The recorded signals were analyzed in more detail with respect to two aspects: the time-dependent amplitude signal and the Fast Fourier Transform (FFT) spectrum of the time signal was analyzed. Thereby, the frequency peak shifts and occurring eigenmodes are identified. To analyze the eigenmodes that occur, a fixed time window of 10 s was shifted over the audio signal and a frequency spectrum was evaluated. This time window is shifted over the entire audio signal without overlap. Only peaks that are larger than the signal’s noise floor by a factor of 2.5 are identified as frequency peaks. The noise floor was calculated as the average amplitude value of the envelope of the frequencies in the range between 10,000 and 15,000 Hz.

### Optical measurement

In order to analyze the damage pattern of the specimens selected for the load increase tests the specimens were examined before and after fatigue testing by means of a Keyence 600 digital microscope (Keyence, Osaka, Japan). For this purpose, an objective with ×20 magnification was used to examine all specimen sides. Areas with a large number of adhesions and fractures were of particular interest here.

## Results and discussion

The goal of this paper is to study cyclic properties of additively manufactured lattice structures and gain a better understanding of the origins of damage accumulation as well as failure mechanisms. As manufacturing caused variations and the effects from geometry overlap, it is very important to study both together to avoid misleading conclusions. A systematic characterization of specimens as a function of their position on the build platform was performed and a subset of specimens selected for both static and cyclic testing based on the results. Throughout the publication, results will be linked back to the basic characterization. In addition, the acoustic emission is used as a source of information.

### Characterization of specimen

**Fig. 3:**
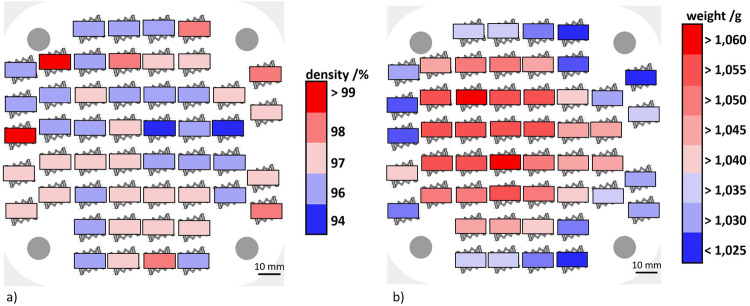
Archimedes measurement results. **a** Density distribution over the build platform, **b** weight distribution over the build platform. (build platform shown in white, where the light gray shading highlights the rounded corners and the dark gray shaded circles in the four corners of the building platform representing the mounting holes).

The Archimedean density measurement allowed the mass and relative density of the samples to be measured. The sample masses vary between 1.0078 g and 1.06143 g with a mean standard deviation of 0.00017 g. The relative densities range from 94.3% to 99% (Gaussian distribution). The sample masses (see Fig. 3a) and the sample densities (see Fig. 3b) are shown as a function of the position of the samples on the building platform. A characteristic pattern emerges for the specimen weights. With a significance of 0.95, the edge samples have a mass between 1.0078 g and 1.04 g. The samples produced in the center of the build platform have a mass between 1.04 g and 1.061 g with a significance of 0.98. However, the maximum of the sample masses is not exactly in the center of the platform, but is slightly shifted to the left^55^. The opposite pattern in weaker expression emerges in the case of relative densities. Higher values tend to occur for samples at the edges of the build platform. The underlying effects will be discussed towards the end of the paper together with the findings from fatigue experiments.

Based on the determined specimen masses six specimens and 16 specimens were selected for the quasi-static and load increase tests, respectively. The remaining specimens were used for a damage study with cyclic experiments at defined load levels and are not part of this work. The selection of specimens is shown in Table 3.

**Table 3:** Purpose of the samples

Series of experiments	Specimen numbers
Quasi-static experiments	10, 51, 52 (low masses) 23, 28, 39 (high masses)
Load increase test	2, 4, 6, 41, 42, 43, 47, 50 (low masses) 14, 15, 17, 21, 22, 26, 27, 29 (high masses)
Targeted adjustment of damage conditions	1, 3, 5, 7, 8, 9, 11, 12, 13, 16, 18, 19, 20, 24, 25, 30, 31, 32, 33, 34, 35, 36, 37, 38, 40, 44, 45, 46, 48, 49

### Quasi-static and load increase experiments

**Fig. 4:**
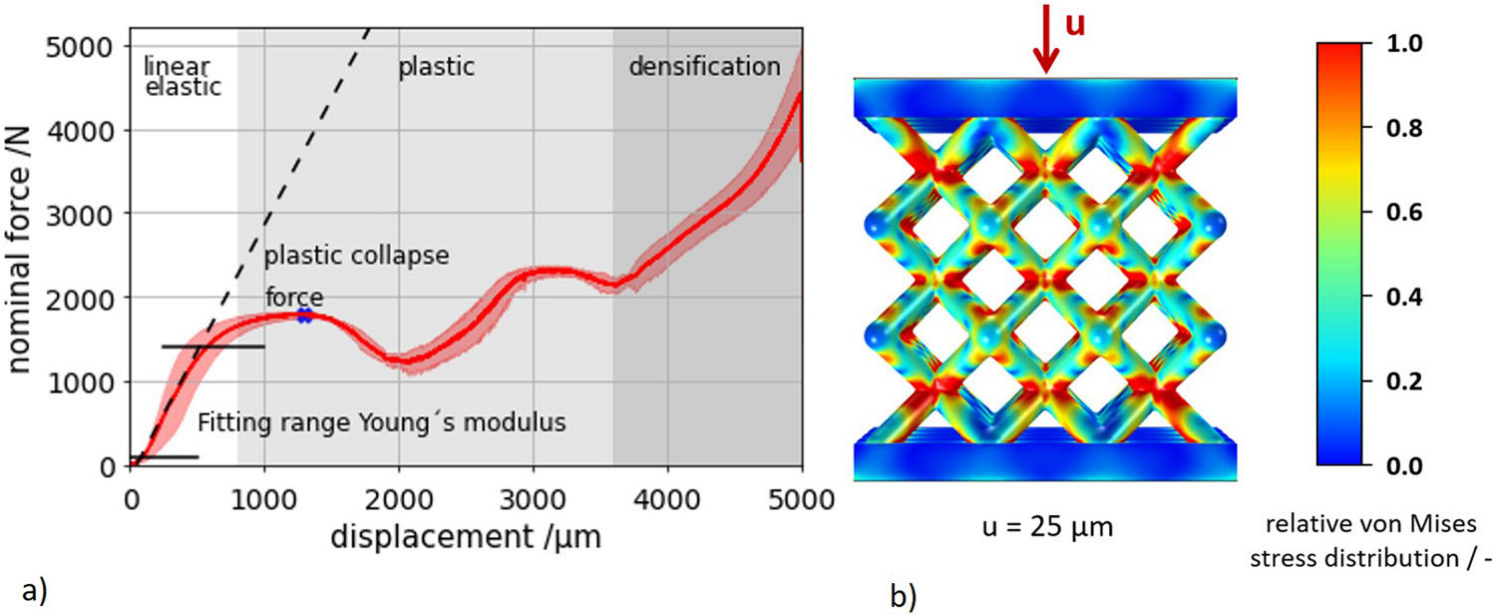
Evaluation of quasi-static compression experiments. **a** Average quasi-static compression test with characteristic lattice structure behavior, with gray shading visually separating the areas of linear elastic behavior, plastic deformation and densification. **b** Numerical simulation of the compression test with local von Mises stress distribution.

The quasi-static properties of lattice structures were evaluated with experiments and simulations. An average, elastic modulus of 459.5 ± 30 MPa and a PCS of 18.9 ± 2.1 MPa were calculated (see Fig. 4a). The determined stiffness of the lattice specimens is comparable to the stiffness values obtained in previous studies on auxetic specimens fabricated with the same fabrication parameters^56^.

**Fig. 5:**
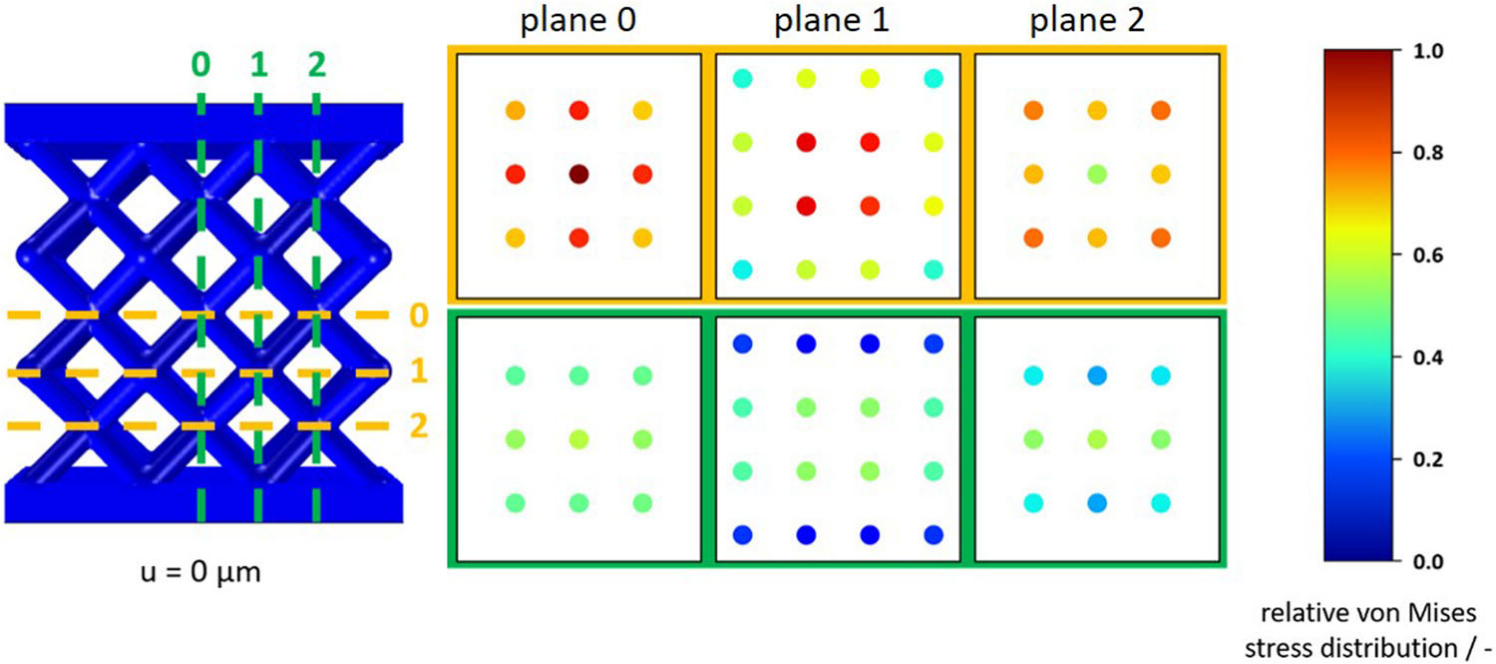
Evaluation of the stress distribution in the lattice nodes by section planes in each lattice plane. (yellow) horizontal section planes 0 to 2, (green) vertical section planes 0 to 2. The von Mises stresses were normalized to the highest present stress.

Although it is known that geometric variations due to manufacturing are to be expected, to complement the quasi-static tests, numerical simulations were performed on an ideal CAD model. The goal was to qualitatively visualize the areas with the highest stresses, which are prone to developing damage. This simulation was performed with small deformations, so it can be assumed that the deformation of the lattice structure is in the linear elastic deformation range. Furthermore, the simulation offers the possibility to illustrate a possible influence of the plates on the stress distribution in the structure. The obtained macroscopic von Mises stress distribution of the structure of 25 µm shows the expected concentration of stresses in the intersection points of the lattice structure (see Fig. 4b). The plates themselves exhibit almost no stresses. It can be concluded that the plates themselves have only minor influence on the mechanical properties of the samples. In order to find the nodes with the greatest load effect, individual cross-sections of the structure were examined in the longitudinal and transverse directions (see Fig. 5) and the mean von Mises stresses were evaluated as the equivalent stress of the individual nodes within the cross-sections. In general, the stress is larger in the transversal than in the longitudinal direction. Further, the inner nodes experience larger stress values except of the node layer next to the plate layer. For this lattice size with 3 × 3 × 3 unit cells, the largest stress value occurs in the middle node of the structure (level 0), which has a high probability of damage initiation based on numerical simulations alone. Based on the quasi-static experiments and simulations, failure is expected to occur starting at the inner nodes and plastic collapse expected around 18.9 ± 0.64 MPa. However, for larger lattice structures, based on a study by White et al.^57^, the inhomogeneities in the stress distribution should decrease asymptotically towards zero with larger number of unit cells, which should be verified in further studies.

### Cyclic experiments

**Fig. 6:**
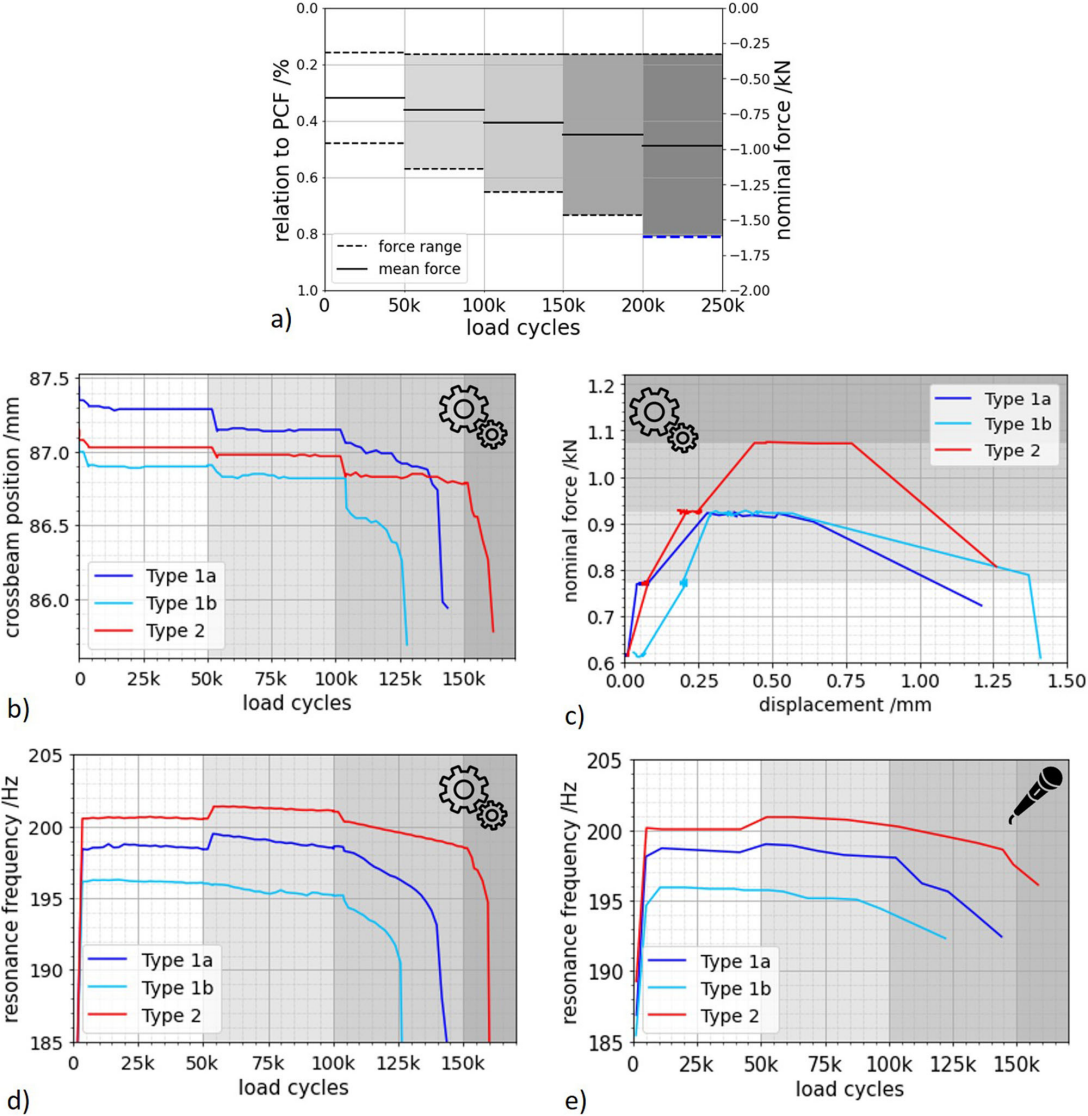
Visualization of the results of the load increase tests, whereby the load stages are separated by different gray shading and icons enable a clear identification of mechanical measurement results (gear wheels) or acoustic measurement results (microphone). **a** Design of the load increase test based on the quasi-static tests and **b** experimental results of the load increase test in particular the position of the crosshead as a function of the number of load cycles, **c** evaluation of the force-displacement curve. Evaluation of the frequencies: **d** resonance frequency of the mechanical testing system over the number of load cycles, **e** evaluation of acousticaly recorded frequency over the number of load cycles.

Cyclic experiments were performed as load increase tests and the load levels determined based on the previous quasi-static experiments (see Fig. 6a). In this section, the results will be presented regarding the load cycles to failure and additionally the machine parameters and acoustic data recorded to understand the damage process before the final failure. Samples failed between ~106,000 and ~164,000 load cycles and no sample reached the 5th load level. The experimental results of all investigated lattice specimens were evaluated in stages by determining the mean and standard deviation of the resonance frequency and the crosshead position for each stage (compare Table 4). It is noticeable that some of the specimens fail at the 3rd load level and some at the 4th load level, which justifies a division into two types. On closer inspection, the specimens that fail in the 3rd load level can be further divided into two subtypes on the basis of the transition from the 1st to the 2nd load level, which is statistically confirmed by Table 4. In general, three different failure scenarios were observed, which are analyzed separately below:

- Type 1a: Failure in load level 3, i.e. higher than 100k cycles (light blue) and continuous transition between load level 1 and load level 2
- Type 1b: Failure in load level 3, i.e. higher than 100k cycles (dark blue) and step-like transition between load level 1 and load level 2
- Type 2: Failure in load level 4, i.e. higher than 150k cycles (red)

**Table 4:** Classification of specimens into types based on the experimental results, taking into account the crosshead position, resonance frequency, and failure stage

	Mean crossbeam position /mm				Mean step size/µm	Mean resonance frequency/Hz				Mean step size/Hz
Stage	1	2	3	4	1→ 2	1	2	3	4	1→ 2
Total	87.0 ± 0.2	86.9 ± 0.18	86.66 ± 0.22	86.75 ± 0.05	−29 ± 12	198.4 ± 1.2	198.6 ± 1.6	194.5 ± 2.2	196.6 ± 1.2	−0.3 ± 0.3
Type 1 average	87.05 ± 0.22	86.89 ± 0.22	86.57 ± 0.27	-	−17 ± 8	197.5 ± 1.5	197.7 ± 2.6	192.3 ± 2.5	-	−0.1 ± 0.8
Type 1a	87.05 ± 0.21	86.90 ± 0.22	86.51 ± 0.22	-	−34 ± 15	197.4 ± 1.8	197.9 ± 2.4	193.2 ± 2.5	-	−0.2 ± 0.1
Type 1b	87.05 ± 0.23	86.89 ± 0.21	86.62 ± 0.31	-	0.0	197.7 ± 1.3	197.3 ± 1.8	191.5 ± 3.5	-	0.0
Type 2	87.00 ± 0.10	86.99 ± 0.11	86.85 ± 0.12	86.75 ± 0.11	−53 ± 35	200.2 ± 0.3	200.4 ± 0.6	198.9 ± 0.5	196.6 ± 1.2	−0.6 ± 0.4

The individual samples can be assigned to these three types with a significance of 0.99. Therefore, exemplary curves for each type are considered in the following. For some of the following analysis, the samples from type 1a and 1b will be averaged as they fail in the same load level.

During the load increase test, the force was increased after defined numbers of load cycles, as described in Fig. 6a). The increase in force is accompanied by a change in the crossbeam position (see Fig. 6b), which led to a macroscopic deformation of the specimens. It can be observed that the specimens of type 1 were compressed more during the transition from stage 1 to stage 2 than the specimens of type 2 with an increment of −34 ± 15 µm and −53 ± 35 µm, respectively. Further compression took place during the transition to load level 3. However, the step was larger than with the first load increase.

Additional information is provided by the force-displacement curve (see Fig. 6c) of the individual specimens. The maximum of these curves shows that the specimens of type 2 can withstand higher forces overall, with the same deformation as the specimens of type 1. In addition, the stiffness of the specimens can be derived from the gradient of the force-displacement curve before reaching the maximum. This clearly shows that the specimens of type 2 have an overall higher stiffness (greater slope) than the specimens of type 1, which show a lower slope.

The observations of the force-displacement data can be substantiated by the resonant frequency shown in Fig. 6d. The resonance frequency of the test system during fatigue provides information about the stiffness of the specimen. Since this is a 3-mass resonance testing system and the weight of the three masses can be assumed to be constant during the test, a change in the resonance frequency is accompanied by a change in the stiffness of the specimen.

The resonance frequencies of the machine exhibited characteristic shapes for the different failure types (Fig. 6d). The type 2 specimens had a higher resonance frequency at the beginning of the experiment than the type 1 specimens with 200.2 Hz and 197.5 Hz respectively (significance = 0.98). This is due to a higher base stiffness (significance = 0.98).

The compression of the lattice changes the notch radii. In addition to this slight geometric change in shape, energy was introduced into the material by the deformation, which according to literature^28^ favors the dislocation movements in the material until the dislocations accumulate at the grain boundaries. These two effects together cause the specimen to be stressed increasing the stiffness of the specimen. This causes a change in the vibration behavior during cyclic loading, which in turn results in an increase in the resonance frequency. The resonance frequency, on the other hand, decreased when considerable macroscopic damage occurred, which ultimately led to the failure of the sample. According to the principle of least constraint, plastic deformation counteracts the distortion of the lattice specimen by dissolving the buildup of dislocations at the grain boundaries. This resulted in a decrease in the stiffness of the specimen.

The time-dependent acoustic signal, which is converted into a spectrum using the FFT, can be used as a further source of information. The resonance frequency of the test system appears as the highest peak in this spectrum. The associated harmonics also appear as peaks. The frequency shift of the highest peak was evaluated as a function of the number of load cycles (Fig. 6e) and was in good agreement with the machine resonance frequency from the testing machine (Fig. 6d). Due to the evaluation effort, however, only 14 measurement points per sample were evaluated here. Two different courses can also be seen in the acoustic data for the specimens of type 1. In principle, it can be said that with a step-shaped frequency increase of up to 0.5 Hz at the transition from the 1st to the 2nd load level, the specimen fails in the 3rd load level. With a frequency increase between 0.7 and 0.85 Hz, the specimen fails in the 4th load level with a significance of 0.96. Thus, a statement about the expected lifetime of the specimen can be made via the magnitude of the step-shaped frequency increase at the transition from load level to load level. In principle, the larger the increase, the higher the remaining life expectancy of the specimen.

### Damage progression and failure prediction

**Fig. 7:**
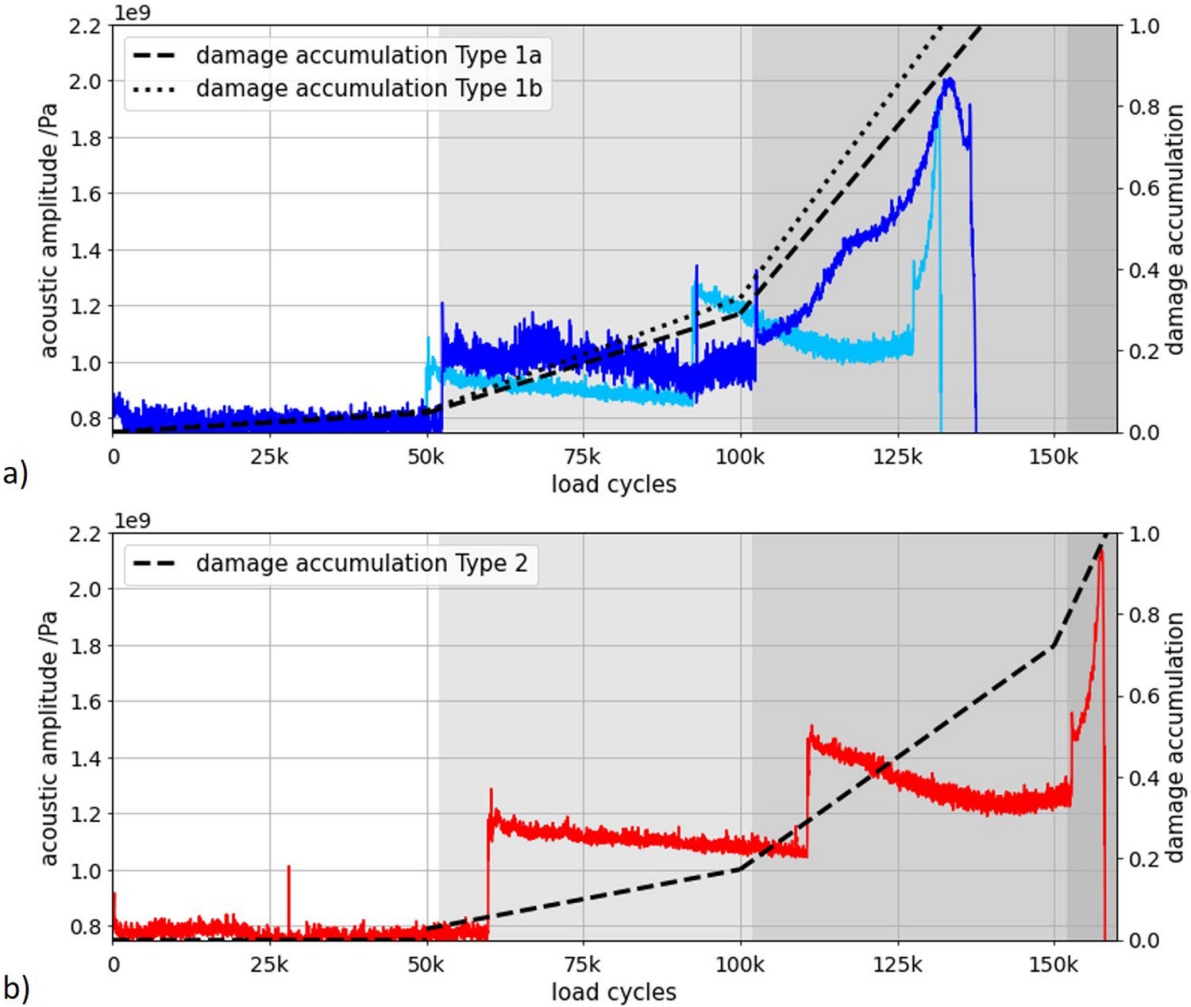
Correlation between the time-dependent acoustic pressure amplitude signal of the acoustic emission measurement and the set damage accumulation, whereby the load stages are separated by different gray shading. **a** Specimens failing at stage 3, **b** Specimens failing at stage 4.

In the next section, results from load increase experiments and acoustic emission data are used to explain the progression of damage in the samples and where the differences in load cycles arise from. To complement experimental data, damage accumulation $D$ estimations were used and calculated as follows^58^:

1 $$D=\frac{{h}_{i}* {\sigma }_{a,i}^{k}}{{\sum }_{i}{h}_{i}* {\sigma }_{a,i}^{k}}$$

where ${h}_{i}$ is the number of load cycles at the particular load level and ${\sigma }_{a,i}^{k}$ is the applied load at the particular load level. The exponent *k* was set to *k* = 6.5 for the alloy used in our study according to Romano et al.^58^. The damage accumulation was calculated for the actual load cycles endured. Three exemplary specimens from the different failure groups were selected, and the acoustic time-dependent amplitude signal evaluated as illustrated in Fig. 7. It can be seen that the amplitude increases with the increasing load cycle number. The time-dependent signals are shown for two specimens that failed at load level 3 (see Fig. 7a) and for one specimen that failed at load level 4 (see Fig. 7b). In order to interpret the signals, the damage accumulation for the respective specimen was also plotted (black). At stage 1, both signals of type 1 and type 2 specimens showed no amplitude change, which according to the damage accumulation indicated only minor damage to the specimens. In stage 2, a small amplitude decrease can be seen. The slope of the signal of the type 1 specimens here was larger than the slope of the signal of the type 2 specimens. The comparison with the damage accumulation showed that for the specimen of type 1 at the end of this loading stage, a damage accumulation of almost 30% is to be expected, while for the specimen of type 2 only a damage accumulation of 20% is to be expected. The larger the amplitude drop in this range, the more damage can be expected. For the type 1 specimen, the cyclic softening led to plastic deformation of the lattice specimen at the transition to stage 3. The amplitude of the acoustic signal increased sharply. The type 2 specimen showed a comparable behavior in load level 3 as in level 2 and did not change into a plastic deformation until load level 4, where the amplitude of the acoustic signal increased due to failure. The comparison of the amplitude signal with the mechanical measured values (cf. Fig. 6) and the damage accumulation shows that the progressive damage of the specimen can be recognized by the amplitude of the acoustic signal. The higher the amplitude, the greater the damage to the specimen. The correlation of the time-dependent acoustic signal with the damage accumulation is quantified by a correlation coefficient of *r* = 0.92354. For a step-like amplitude increase of 18–25% at the transition from the 1st to the 2nd loading stage, failure of the specimen occurs at the 3rd loading stage with a significance of 0.92. With an amplitude increase of approx. 40-50%, the specimens fail with a significance of 0.95 in the 4th load stage. The size of the step-shaped amplitude increase at the transition from one load level to the next can therefore be used to make a statement about the expected lifetime of the specimen. In principle, the larger the step, the higher the remaining life expectancy of the specimen. The point in time at which the amplitude increases in steps also contains information. A trend could be identified according to which the specimens fail earlier, the earlier the step-like increase occurred. It is noticeable that the specimens of type 1b, which at the beginning of the test had the lowest machine resonance frequency of 197.4 ± 1.8 Hz and thus the lowest stiffness of the specimens, showed this step already after ~48,000 load cycles. For the specimens of type 2, which had a higher resonance frequency of 200.2 ± 0.3 Hz, this step-like increase in amplitude occurred only after about 60,000 load cycles. While damage accumulation can only be meaningfully calculated after specimen failure, the acoustic signal offers the possibility of damage prediction based on the amplitude increase of the signal.

**Fig. 8:**
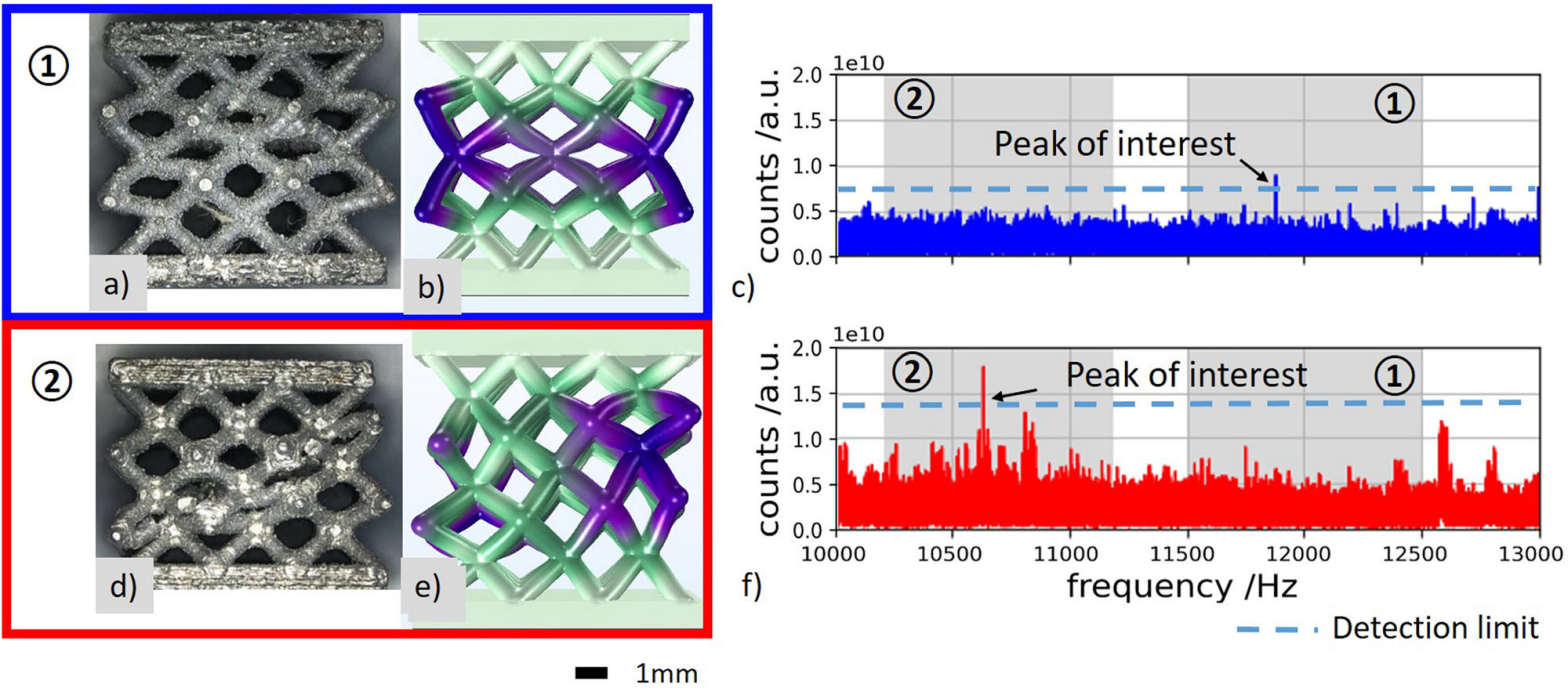
Qualitative correlation between damage pattern and eigenmodes based on the specific eigenfrequency peaks occurring in the acoustic frequency spectrum. **a** Optical micrograph of damage pattern type 1, **b** eigenmode type 1 (green areas of the lattice show only slight deformations, while purple areas undergo higher deformations), **c** natural vibration at 11,820 Hz leads to damage pattern type 1 (the numerically determined expectation ranges of the natural vibrations are highlighted in gray), **d** optical micrograph of damage pattern type 2, **e** eigenmode type 2 (green areas of the lattice show only slight deformations, while purple areas undergo higher deformations). **f** Natural vibration at 10,690 Hz leads to damage pattern type 2 (the numerically determined expectation ranges of the natural vibrations are highlighted in gray).

After the fatigue tests, the specimens of the two failure types were examined by light microscopy to show the macroscopic failure scenario (Fig. 8a, d). The failure type 1 specimens showed considerable compression and buckling with the failure of several struts. For the failure type 2 specimens, multiple struts failed, resulting in 45° of shear.

To get an understanding of the different failure types, an eigenmode simulation of the first 25 eigenmodes was performed on the lattice specimen for a range of Young’s modulus. For this purpose, the Young’s modulus was varied based on the values determined in the quasi-static compression tests between 372.3 and 573.6 MPa. Two eigenmodes with a qualitative analogy to the failure patterns that occurred were empirically identified (see Fig. 8b, e). The first eigenmode occurs between 11,500 and 12,500 Hz and shows no deflection of the plates and considerable deformation of the sample. The inner layers of the unit cells are compressed the most, and the outer unit cells of the middle lattice planes are pushed outward. The second eigenmode occurs between 10,200 and 11,150 Hz and exhibits a shear-like deformation with the most deformed unit cells along the 45° shear diagonal.

The cyclic loading of the specimens during the test excited not only the system-specific frequencies (Fig. 6c), but also the natural frequencies of the lattice specimens. It is well known from the literature that geometry and mechanical properties affect the natural frequencies of structures^30^. In the next section, the emitted acoustic spectrum is analyzed with respect to the differences between the two failure modes, with an emphasis on the frequency ranges determined above.

For specimens with type 1 failure, natural frequencies with a frequency between 11,500 and 12,500 Hz could be identified after 84.7 ± 3% of the total load cycles with a significance of 0.998 (see Fig. 8c). This corresponds to the beginning of the considerable decrease of the resonance frequency due to the plastic deformation of the lattice structure in load level 3. In the range from 10,200 to 11,150 Hz, no peaks with amplitude higher than 2.5 times the noise floor were detected.

The damage pattern of specimens failing in type 2 resembles an eigenmode in the frequency range from 10,200 to 11,150 Hz (see Fig. 8f). Again, the corresponding peak in the spectrum from the onset of compaction could be visibly detected after 86.6 ± 3% of the total load cycles endured with a significance of 0.996. In the range from 11,500 and 12,500 Hz, no peaks with amplitude higher than 2.5 times the noise floor were detected. Due to the fact that these frequencies only appeared when the lattice specimens were deformed, it can therefore be assumed that certain eigenmodes, which have a similar shape to the damage phenomenon, are preferentially excited by the initiation of damage to the specimens.

**Fig. 9:**
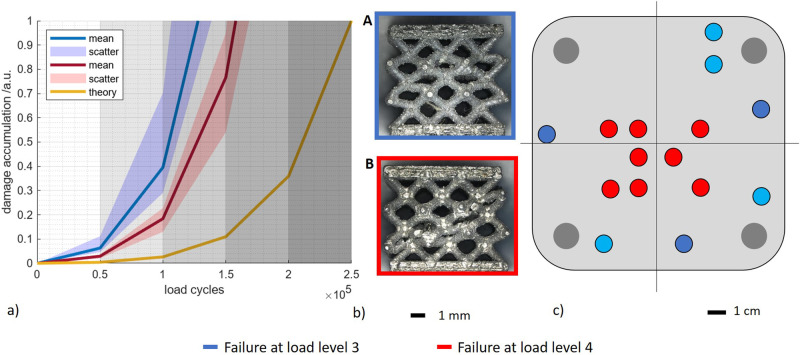
Summary of the fatigue properties of the specimens differentiating the two observed failure types: type A (blue) and type B (red). **a** Assessment of the sample damage based on the adjusted damage accumulation (gray shading visually separates the individual load levels from each other), **b** optical micrographs of the two failure types A (top) and type B (below), and **c** the damage pattern in correlation with the build platform position.

**Fig. 10:**
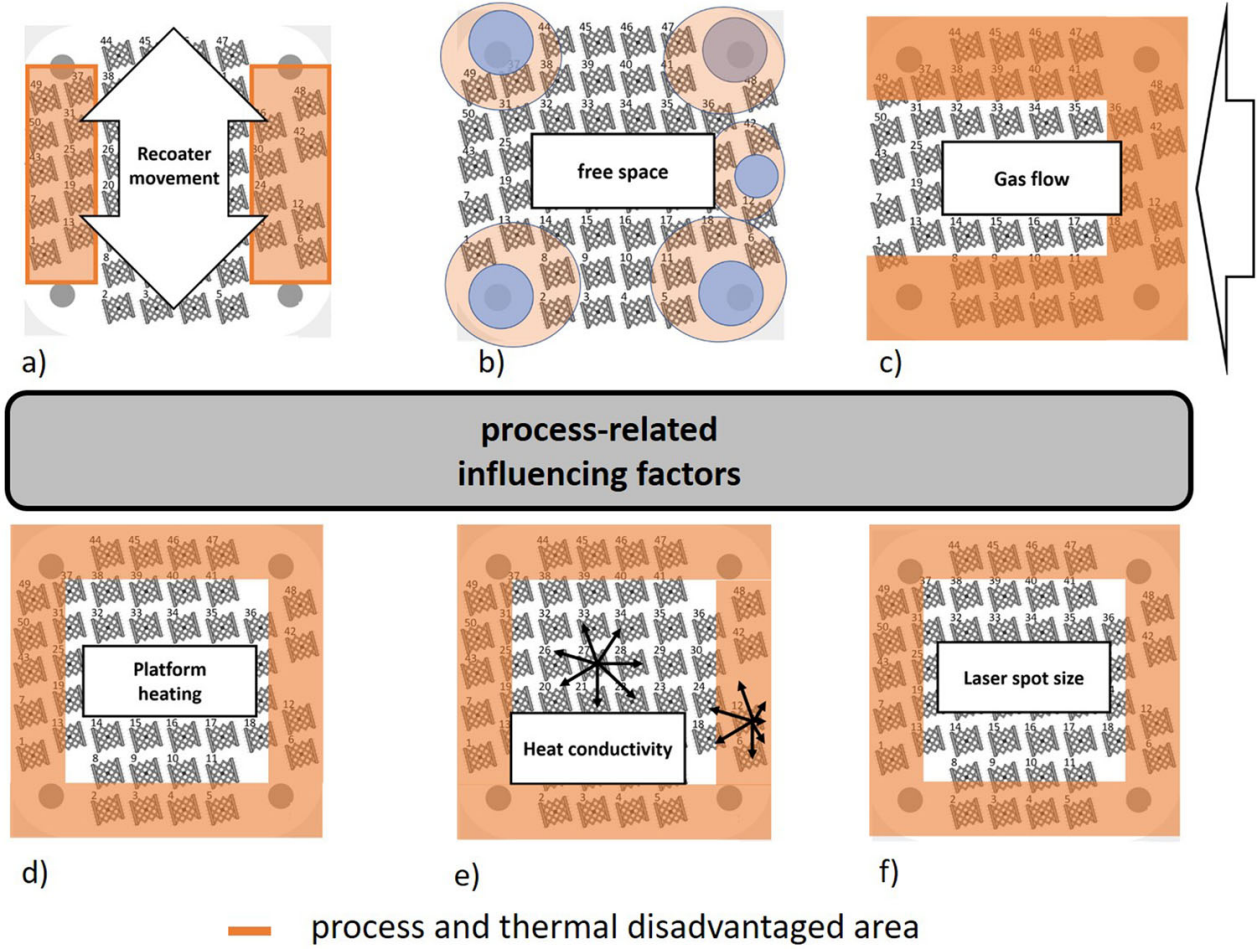
Process-related factors that influence the properties of the samples depending on the position of the build platform, with the influenced areas indicated by orange shading. **a** Recoater movement (arrow shows the direction of the gas flow, up and down), **b** Free space (marked with blue circles), **c** gas flow direction (arrow shows the direction of the gas flow from right to left), **d** Platform heating, **e** Heat conduction (arrows show the direction of heat dissipation from the sample), **f** laser spot size.

In summary, the damage accumulation for the two groups was evaluated and presented in Fig. 9a. In addition to the damage accumulation curves fitted to the individual specimens, the ideal curve for failure at the end of the planned load increase test was also added. These curves show that the expected damage accumulation deviates from the experimentally determined damage accumulation, since the calculation assumes ideal structures that do not exist in reality. However, the division into two separate groups is clearly visible. The less stiff specimens showed earlier damage development due to the cyclic loading. While these specimens were expected to reach 40% damage accumulation at 100,000 load cycles, this damage does not occur until about 120,000 cycles for the stiffer specimens. This shows the deviation of the real specimens from an ideal specimen, where this degree of damage would not occur until about 200,000 load cycles. Considering the damage accumulation provides the explanation of why the stiffer specimens endure greater load than the specimens with a lower stiffness. It can be assumed that on the one hand, the stiffness, and on the other hand, the higher load of the specimen influenced the vibration of the specimens, so that a characteristic acoustic signal pattern per type can be recognized. In addition, the determined properties were analyzed as a function of the position on the building platform (cf. Fig. 9c). The Type 1 specimens (blue) are located in the peripheral zones of the platform. The type 2 specimens (red) were produced in the center of the build platform. This clearly shows that the position on the build platform determines the stiffness of the specimens, on which the achievable load level and failure behavior depend. It is noticeable that specimens placed directly on the mounting bolts do not exhibit this stiffness increase (see Fig. 6). These specimens are softened during the load increase testing. In order to explain why this effect occurs with these specimens in particular, manufacturing-related influencing factors must be considered in more detail (see Fig. 10). Microscopic analysis of the damage shows struts breaks just above the lattice nodes on the top of the specimens (see Fig. 9b). A simulation was used to further localize the damaged areas.

It is noticeable that specimens positioned directly on the mounting bolts (light blue) do not exhibit this increase in stiffness (see Fig. 6). These specimens are softened cyclically throughout the test run. In order to explain why this effect occurs with these specimens in particular, manufacturing-related influencing factors must be considered in more detail (see Fig. 10). Microscopic analysis of the damage pattern revealed struts breaks just above the lattice nodes on the upskin side of the specimens. Based on the presented numerical simulations of the stress distribution within the lattice specimen, the probability of crack initiation is greatest at this node. However, the simulation does not include the manufacturing-induced powder deposits located on the side of the specimen facing away from the laser. These support the nodes, so that the stress peak shifts to the upskin side of the specimen and explain the failure scenarios observed in the experiments.

### Process-related classification of the property distribution

The evaluation of the previous tests shows a ring-shaped property distribution of the 16 lattice samples tested. In order to ensure reproducibility of the measurement results (see 1 Supplementary results and discussion), a second series of experiments was carried out with an equivalent setup. The classification of the samples into types 1 and 2 and the ring-shaped distribution on the building platform were confirmed using supplementary Table 1 and supplementary Fig. 1. Six different process-related influencing factors (see Fig. 10) were investigated with regard to their effect on the property distribution as a function of the build platform.

Due to the circular distribution, the influence of the recoater movement, which would cause a property distribution as in Fig. 10a, can be excluded. To counteract this influence preventively, the test specimens were arranged rotated by 15° to the coater orientation during the planning of the construction measure. This prevents the recoater from sticking to the specimens.

The influence of the gas flow can be seen most clearly from the distribution of the specimen masses on the build platform (see Fig. 10c). The gas flows linearly from right to left across the build platform, forming a characteristic flow velocity distribution. In their study, Reijonen et al.^55^ found that the flow velocity basically decreases from right to left. This effect is additionally superimposed by a kind of flow around the building platform, whereby the flow velocity in the center of the platform is lower than at the upper and lower edges. The flow velocity causes a selection of the powder according to the particle size. The higher the velocity, the more and larger particles can be moved. This causes particles to be moved from the right side of the build platform, so there is less powder to be melted. The specimens positioned there have lower masses. The blown particles accumulate slightly to the left of the center, which explains the higher masses in this area.

The factors (Fig. 10d–f) with their resulting property distribution, on the other hand, can be clearly assigned to the experimentally determined distribution. In order to clearly identify platform heating as an influencing factor, the heat distribution of the heated build platform in the build space of the SLM machine was measured using a non-contact thermometer. It was found that the platform is uniformly heated to 150 °C in the area inside the fastening screws. The peripheral areas, however, show a temperature difference of 15 °C. In addition to this temperature gradient, the heat can be dissipated differently from the specimens, depending on their position on the build platform (see Fig. 10e). In the center of the build platform, heat dissipation is equally possible in all directions, whereas in the edge areas, heat dissipation is less pronounced toward the edge than toward the center. In addition, the specimens are arranged most densely in the center of the platform. If these factors are considered together, it can be seen that the temperature gradient is greater in the edge zones of the building platform than in the center of the platform. This causes the molten metal to cool more rapidly, which further promotes the process-related preferential orientation of the microstructure grains. Long stem crystals^6,59,60^ can form along the preferred direction. A lower temperature gradient is present in the center of the build platform, which causes the molten metal to cool more slowly. Thus, a microstructure with preferential orientation can still form, but to a smaller extent. The additional heat treatment connected to the manufacturing process serves to reduce the internal stresses in the specimens but is not sufficient for homogenization of the microstructure^6,60,61^. Another factor influencing the property distribution is the positioning of the laser (Fig. 10f) centrally above the build platform. The laser spot is the smallest in the center of the build platform. The laser beam reaches the outer areas by deflection with the help of optics, which leads to a widening of the laser spot^62^. This causes a reduction in the point energy density of the laser beam, which makes it more difficult to melt the powder.

As can be seen in Fig. 10b, there are areas on the build platform where either no test specimens can be positioned (platform fastening screws) or have been placed. In these areas, there are no specimens during the manufacturing process, only powder, which has an isolating effect. In combination with the steel screws used to fasten the build platform, heat buildup occurs in these areas, causing the neighboring specimens to undergo additional heat treatment during the manufacturing process. Accordingly, the melt cools more slowly, which leads to the growth of the mixed crystal grains and thus to a coarser microstructure^63^. The findings of Takata et al.^64^ on the influence of heat treatment on the microstructure of manufactured L-PBF-AlSi10Mg structures further substantiate the observations. Several studies show that a coarser microstructure is associated with poorer resistance to cyclic loading^32,65^. The coarser the microstructure, the fewer grain boundaries are available at which dislocations can accumulate. This effect provides an explanation for the continuous softening of these specimens under cyclic loading. In addition, it is known that poorer heat dissipation in the specimen leads to more powder buildup, which reduces the relative density of the lattice structure^51^. All other specimens show an increase in stiffness at the transition from load level 1 to load level 2 (see Fig. 6d).

If the influences of these factors are considered together, the distribution described above can be explained across the building platform of the properties of the lattice specimens. The lattice specimens in the center of the platform show a higher stiffness, which is due to a more homogeneous and finer microstructure than in the edge specimens. The finer the microstructure, the more dislocation motion is hindered at the grain boundaries, increasing the stiffness^61^. The damage pattern can also be explained in terms of stiffness differences. With higher stiffness of the lattice specimens, bending-dominated damage occurs; with lower stiffness, the damage is stretch-dominated^1^. However, besides the influence of the microstructure on the mechanical properties of the lattice specimens under cyclic loading, the porosity should also be considered. Based on the study by Reijonen et al.^55^, the porosity in the edge areas of the build platform should be lower than in the center of the build platform due to the higher flow velocities of the shielding gas. In further studies, this aspect should be examined in more detail for thin-walled structural parts.

### Limitation of the study

In this study, the fatigue behavior of bcc lattice structures was investigated in order to identify possible failure initiation points and to be able to classify the experimental results with regard to the influencing factors of geometry, plates, and manufacturing influences, a numerical simulation was used (see Fig. 5). For this purpose, a purely qualitative analysis of the stress distribution within the lattice structure at very small deformations was carried out. An ideal CAD model of the lattice specimen was implemented as the geometry. In order to keep the effort-benefit ratio in balance, a quasi-static compression test was simulated, as this could be simulated with a manageable computing time. As this is a purely qualitative analysis of the stress distribution within the geometry at very small deformations, this simulation can be carried out independently of a material. For the purpose of completeness, the material parameters of the alloy considered in the study were included in the model. The consideration of an ideal lattice provides sufficient information to validate the experimental results. A specific localization of the stress peaks that occur due to production-related geometric inaccuracies cannot be carried out with this simulation. However, the simulation can be used to recognize stress increases at the lattice nodes, which can also be assumed for cyclic loads.

In addition to these geometrically induced stress increases, microstructural changes within the material also lead to failure of the samples under cyclic loading. These could be assigned to the experimental observations on the basis of literature sources, but were not verified experimentally.

Optimized L-PBF parameter sets for AlSi10Mg lattice structures as used in this study exclude a direct transfer to other materials and their manufacturing-related properties. Due to different compositions in loading conditions interacting with manufacturing-related properties, the present results cover bcc lattices only. Further transferability to other lattice types and signatures to estimate the behavior of a novel structure based on an investigated lattice needs clarification in future study.

## Conclusions

In this article, the fatigue behavior of truss lattice structures made of AlSi10Mg produced by L-PBF was analyzed. It has been shown that a characteristic distribution of specimen properties emerges due to the L-PBF- machine used. This distribution is due to the process-related influencing factors, such as the gas flow, the specimen placement on the platform, the heat conduction, the platform heating and the laser spot size. The specimens fabricated at the edge of the platform exhibit lower stiffness and fail earlier than the specimens fabricated in the center of the build platform. The mechanical fatigue tests were accompanied by acoustic emission monitoring. It was found that the data showed a strong correlation with the mechanical data. Analyzing the acoustic signals in terms of the time-dependent amplitude signal and the frequency spectrum, it was found that the life expectancy of the specimens for this load can be predicted with a significance of 0.95 after 50,000 load cycles. This means that acoustic emission monitoring offers a high degree of correlation with the mechanical data. Thus, acoustic emission monitoring offers the possibility to estimate the expected lifetime already during the running test and to predict the failure type. This offers the chance for in situ monitoring of the specimens for industrial applications. This statement should be validated in further studies by examining a larger sample set and systematic variations of process parameters. The study can also be extended towards different materials and by selecting more specific microphone positions based on the knowledge gained. The use of a microphone with a wider frequency response or piezoelectric transducers could also allow the detection of higher resonance frequencies and the resolution of specific eigenmodes, since their effect is more pronounced at higher frequencies. Additional information on the damage behavior of the specimens could be obtained by observing specifically set loading conditions.

### Supplementary information

Supplementary Information

## Data Availability

The data that support the findings of this study are available from the corresponding author upon reasonable request.
