# Supplementary material for: Acoustic emission of lattice structures under cycling loading relates process parameters with fatigue properties
Source: Commun Eng. 2024 Mar 22;3:56. doi: 10.1038/s44172-024-00196-2 (PMC10959972; doi:10.1038/s44172-024-00196-2)
Supplement: Supplementary file 1 — Supplementary Information [file 44172_2024_196_MOESM1_ESM.pdf]

1. Supplementary results and discussion

To check the reproducibility of the results, the series of measurements was repeated with specimens independently manufactured based on the process and material parameters from the paper on the same LPBF machine. The crosshead position and the test frequency of the 3-mass oscillator were analyzed as described in section results and discussion. In Table S1, the evaluation of the 1st series of measurements (as listed in main paper) was compared with the evaluation of the 2nd series of measurements. The measured values show only minor deviations between the two measurement series. From this, it can be concluded that the findings are comparable.

Table S1 Comparison of the results for two independently manufactured specimen sets. Specimen set (1) was already discussed in the paper and specimen set (2) are new results. The specimens were first analyzed all together and then classified into types 1a, 1b and 2 according to the criteria described in the paper. Analysis takes into account the crosshead position, resonant frequency and failure stage.

	Mean crosshead position /mm				Mean step size /µm	Mean resonance frequency /Hz				Mean step size /Hz
Stage	1	2	3	4	1→ 2	1	2	3	4	1→ 2
Total (1)	87.03 ±0.18	86.88 ±0.18	86.66 ±0.22	86.75 ±0.05	29 ±12	198.4 ±1.2	198.6 ±1.6	194.5 ±2.2	196.6 ±1.2	-0.3 ±0.3
Total (2)	87.14 ±0.10	86.57 ±0.18	86.67 ±0.22	86.70 ±0.05	60.3 ±12	198.4 ±1.1	198.5 ±1.6	194.5 ±2.1	196.2 ±1.1	-0,3 ±0.3
Type 1 average (1)	87.05 ±0.22	86.89 ±0.22	86.57 ±0.27	-	-17 ±8	197.5 ±1.5	197.7 ±2.6	192.3 ±2.5	-	-0.1 ±0.8
Type 1 average (2)	87.05 ±0.22	86.83 ±0.22	86.56 ±0.26	-	-36 ±12	197.5 ±1.6	197.6 ±2.5	192.3 ±2.5	-	-0.1 ±0.2
Type 1a (1)	87.05 ±0.21	86.9 ±0.22	86.51 ±0.22	-	-34 ±15	197.4 ±1.8	197.9 ±2.4	193.2 ±2.5	-	-0.2 ±0.3
Type 1a (2)	87.05 ±0.22	86.78 ±0.23	86.52 ±0.22	-	-36 ±12	197.4 ±1.8	197.9 ±2.4	193.2 ±2.5	-	-0.2 ±0.3
Type 1b (1)	87.05 ±0.23	86.89 ±0.21	86.62 ±0.31	-	0.0	197.7 ±1.3	197.3 ±1.8	191.5 ±3.5	-	0.0
Type 1b (2)	87.06 ±0.23	86.88 ±0.21	86.61 ±0.32	-	0.0	197.7 ±1.3	197.3 ±1.7	191.5 ±3.5	-	0.0
Type 2 (1)	87.00 ±0.10	86.99 ±0.11	86.85 ±0.12	86.75 ±0.11	-53 ±35	200.1 ±0.3	200.4 ±0.6	198.7 ±0.5	196.6 ±1.2	-0.6 ±0.4
Type 2 (2)	87.01 ±0.10	86.99 ±0.11	86.86 ±0.12	86.70 ±0.05	-52 ±38	200.1 ±0.3	200.4 ±0.6	198.8 ±0.5	196.2 ±1.1	-0.6 ±0.4

In addition to this evaluation, the damage patterns of the samples from the 2nd construction job were also analyzed (see Figure S1). The two different damage patterns presented in above paper were observed and the damage patterns of the samples were related to the position of the sample on the building platform during the manufacturing process (Figure S1). Like the samples of the first build job, the samples at the edge of the build platform fail according to damage pattern A and the samples in the middle of the build platform fail according to damage pattern B. In summary, it can be concluded from this 2nd series of measurements that a cross-build job reproducibility could be demonstrated using an identical manufacturing process.

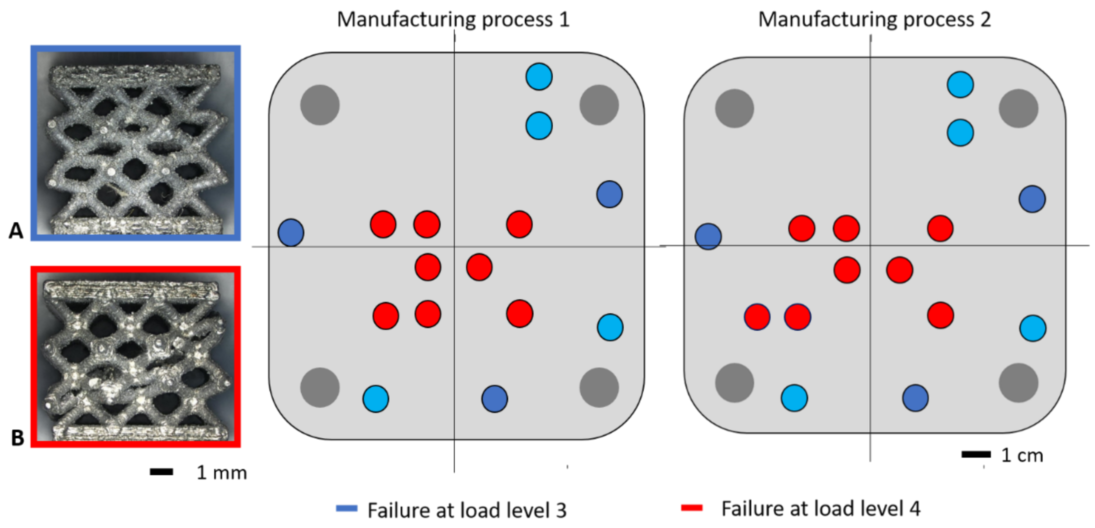

Figure S1. Comparison of the damage patterns of the lattice specimens from two different manufacturing processes and classification of these in relation to the respective building platform position during the manufacturing process.
